# Evolution of Child and Youth Mental Health in the Context of the COVID-19 Pandemic: A Longitudinal Analysis

**DOI:** 10.3390/children11060660

**Published:** 2024-05-28

**Authors:** Arancha Bernal-Jiménez, Xosé Ramón García-Soto, Sara Calvo-Simal, Yolanda Álvarez-Férnandez, Rocío Gordo-Seco, M. Rosario Hernando-Segura, Ángela Osorio-Guzmán, Ana Gentil-Gutiérrez, Jessica Fernández-Solana, Jerónimo Javier González-Bernal, Josefa González-Santos

**Affiliations:** 1 Psychiatry Service, University Hospital of Burgos, 09006 Burgos, Spain; abernalj@saludcastillayleon.es (A.B.-J.); xgarcia@saludcastillayleon.es (X.R.G.-S.); yalvarezf@saludcastillayleon.es (Y.Á.-F.); rgordo@saludcastillayleon.es (R.G.-S.); rhernandos@saludcastillayleon.es (M.R.H.-S.); aosorio@saludcastillayleon.es (Á.O.-G.); 2 Biostatistics Unit of the Research Department, University Hospital of Burgos, 09006 Burgos, Spain; scalvo@hubu.es; 3 Department of Health Sciences, University of Burgos, 09001 Burgos, Spain; agentil@ubu.es (A.G.-G.); jejavier@ubu.es (J.J.G.-B.); mjgonzalez@ubu.es (J.G.-S.)

**Keywords:** COVID-19, mental health, children, youth, internalizing symptoms, externalizing symptoms, GAF, global functioning

## Abstract

Background: The COVID-19 pandemic generated uncertainty and disruption among the child and adolescent population. Multiple studies have documented a worsening of mental health following the pandemic. The main objective of this longitudinal study is to analyze the short-, medium-, and long-term evolution of the overall functioning of children and adolescents treated by a child and adolescent mental health team in the context of the COVID-19 pandemic. Methods: 420 patients aged 3 to 18 were assessed using the Global Assessment of Functioning (GAF) scale at three time points: during the lockdown, three months later, and three years later. Differences based on gender, diagnosis, and time were analyzed. Results: A significant improvement was observed in the short-term (three months) and long-term (three years) compared to the lockdown period. This improvement was maintained in all diagnostic subgroups except for mixed cases (severe mental pathology), which showed the least improvement. No significant differences were found between males and females. Conclusions: The child and adolescent population showed a greater capacity for adaptation to the lockdown than expected. Family support, decreased stress, and therapeutic intervention appear to have played an important role in improving mental health.

## 1. Introduction

The COVID-19 pandemic became an unprecedented disruptive event in people’s lives globally, including the child and adolescent population [1,2]. From a child’s perspective, they faced multiple developmental challenges during this period. One of the most prominent was the abrupt disruption of their usual routines due to lockdown. The lack of social interaction with their peers in the school environment limited their social and emotional development by depriving them of crucial experiences for their learning and growth [3]. Likewise, limited contact with other children and the ubiquitous presence of masks hindered oral communication, preventing the proper visualization of sounds and facial expressions, essential elements for language acquisition [4,5].

Thus, during a critical period of their psychological development, this age group experienced a significant impact due to social isolation measures, disruption of school and family routines, and widespread uncertainty [6,7]. The distinctive characteristics of childhood and adolescence development make this population particularly vulnerable to the effects of stress and adversity. During this stage, children and adolescents are in the process of constructing their identity, developing social and emotional skills, and learning strategies to cope with difficulties [8]. However, the pandemic altered the normal circumstances of these developmental processes and raised doubts about the outcome of these developmental processes and raised doubts about the outcome of the experience.

The emotional and social impact of the COVID-19 pandemic on children’s mental health is still unknown, but various studies anticipated a negative psychological effect [9,10,11]. During the lockdown and the immediate post-lockdown period, studies identified several issues in the child population, such as sleep disorders, feelings of loneliness, anxiety, depression, hyperactivity, post-traumatic stress, irritability, challenging behaviors, fear of illness, nightmares, loss of appetite, physical discomfort, agitation, lack of attention, attachment problems [12,13], and hyperactivity [14,15]. The first study published in Chile during the lockdown confirmed symptoms of negative impact on adults’ mental health during the COVID-19 quarantine [10,16], while a study conducted with preschool and school-age children who had certain vulnerabilities observed a significant increase in symptoms compared to the pre-pandemic period [17]. Hence, concerns for this group and the risks to their mental health arose early [18]. Likewise, a very striking article warned about the risks faced by minors in an environment where stress and anxiety levels are high and where there has been a significant disruption of their routines and global functioning [19].

In most of the studies, an increase in levels of anxiety and depression in the child and adolescent population during the pandemic compared to previous periods was observed [20,21,22,23,24,25]. This impact was also evidenced in a meta-analysis that examined a total of 334 research studies addressing the subject; out of these, fourteen were included in the review, as they were the only ones that met the established criteria: original research studies, with a prospective methodological design, published from the year 2020 onwards, and whose results evaluated levels of depression, stress, anxiety, and/or behavioral problems using scales in the child and adolescent population during the SARS-CoV-2 pandemic [26]. The authors posited in their conclusions that internalizing symptoms (anxiety, depression, and emotional problems) significantly increased following the pandemic, in contrast to externalizing symptoms.

However, there is another interpretative approach to the situation that, without denying the risks, also considers the potential benefits of the situation. Spending time with family during lockdown may have generated several positive effects that helped mitigate the negative impact of lockdown. According to several researchers, the fact that families spent more time together, without the social pressures of daily life, such as school and work routines, could have resulted in increased positive interactions and led to improved communication and a deeper understanding of the needs and feelings of each family member [27,28,29].

Based on this approach, our preliminary research did not find significant psychopathological effects of the COVID-19 lockdown [30]. In this study, a prospective investigation was conducted on the mental health of 422 children and adolescents aged 3 to 18 years during the months of March to May 2020. During this period, patients were monitored via telephone at the Child and Adolescent Mental Health Services (CAMHS), with their symptoms, diagnoses, and overall functioning levels recorded using the Global Assessment of Functioning (GAF) scale in each review appointment. The GAF scores obtained before and during the lockdown were statistically compared, suggesting that the mental health status of the patients did not worsen following the onset of the COVID-19 lockdown. Neither worsening stages nor significant differences in the reported symptoms were observed during these periods.

Aware of the significant challenge faced by the child and adolescent population in situations like the COVID-19 pandemic, this longitudinal study is being conducted, hypothesizing that lockdown situations will not necessarily harm the clinical status of children and adolescents if a specific guidance program and individualized attention to families are applied, and it may even allow ongoing therapeutic programs to achieve short-, medium-, and long-term improvements. For this reason, our CAMHS has prepared a model of proto-colonized telephone care during the lockdown due to COVID-19 with the following objectives: to maintain the psychological assistance of patients, provide telephone consultations, systematically collect information on the situation and needs of families, record the state of mental health of patients, and offer support information to families to manage the difficulties caused by the lockdown. In this way, the present study aims to analyze the short-, medium-, and long-term evolution of the clinical status of children and adolescents treated in a CAMHS during the lockdown period in Spain amid the COVID-19 pandemic.

## 2. Materials and Methods

### 2.1. Study Design and Participants

This present study was conducted at the CAMHS of the University Hospital of Burgos, a specialized service that is integrated into the Spanish public health system and is engaged in the care and treatment of children and adolescents with mental health problems. These are outpatient care units that receive patients from primary care and various specialized care services. The CAMHS works in collaboration with other health professionals, such as pediatricians and neurologists, as well as with educational centers and social services, to guarantee comprehensive care for their patients.

A longitudinal and prospective study of a cohort of children and adolescents treated at the CAMHS consultations in Burgos, located at the University Hospital of Burgos (HUBU), during the COVID-19 lockdown period was presented. The minors were already receiving treatment at the unit before the pandemic outbreak and were followed during and after the lockdown. As the main objective of the CAMHS was to maintain psychological assistance during the lockdown, inclusion criteria considered for participation in the program included all patients currently under a follow-up who had signed the corresponding informed consent. 

The research plan, including protocols for non-face-to-face care, was submitted to the Medical Research Ethics Committee of the Burgos Health Area before the start of the lockdown and obtained the required authorization with registration number CEIm 2293. Data collection began in the early days of the lockdown after obtaining informed consent from the participants (in this case, through parents, mothers, or legal guardians), ensuring their voluntary participation and anonymity. The data were collected by the mental health teams following previously agreed upon telephone care protocols and were presented to the Medical Research Ethics Committee. The data were anonymized to ensure patient confidentiality. The data processing complied with the European General Data Protection Regulation and Organic Law 3/2018 on the Protection of Personal Data and the guarantee of digital rights.

### 2.2. Procedure

The health status of 420 patients aged between 3 and 18 years was evaluated through clinical information collected by the various practitioners within the CAMHT. The participants were patients who were already under a follow-up at the beginning of the lockdown and requested care during it.

To determine the severity of psychiatric symptoms and disorders, the GAF scale was used. This assessment was conducted at each consultation during the lockdown period (March–May 2020), three months after the end of the lockdown (September 2020), and in a follow-up evaluation at three years (September 2023). These time points were deemed appropriate, considering that if the lockdown had impacted the mental health of children and adolescents, it would be reflected in the emergence of emotional or behavioral symptoms within three months after the stressful event and/or in the worsening or exacerbation of premorbid pathology. The latter assessment was conducted in person for patients under a follow-up at the unit at the time of evaluation and by telephone for those who had been discharged.

The interviews carried out during and after the lockdown were conducted by telephone in a protocolized procedure with an average duration of 60 min. In these telephone calls, the corresponding professional was to collect information on the general situation of the family (number of people living in the home, type of household, and risk of exposure to COVID-19), the main difficulties identified at the time, and the mental health status of the children. The mental health of the patients was also assessed by means of an individualized clinical interview and a psychopathological examination using a symptom checklist (attentional difficulties, behavioral problems, emotional problems, social relationship difficulties, and somatic symptoms).

In addition, these interviews also provided guidelines for action regarding the general situation of lockdown, specific orientations for each clinical case, and, in those cases where necessary, the corresponding treatment was carried out (crisis intervention, medication control, and individual psychotherapy).

Additionally, families were provided access to a blog where daily content with guidelines for families was published (blog: https://ramonsotoinfancia.wordpress.com/ accessed on 18 May 2020). General information related to the lockdown, organizational problems, and other concerns expressed by families were provided, along with specific guidance on the child’s psychopathological situation, including psychological and pharmacological treatment. Weekly articles were also published in the local press, and interventions were made on local television. Subsequently, after the end of the lockdown, individualized psychotherapeutic interventions adapted to each clinical case were conducted in person.

### 2.3. Instruments

Demographic data such as the age, sex of the participants, and the diagnosis issued were collected. Subsequently, diagnoses were grouped into internalizing symptoms, externalizing symptoms, mixed presentations, and undiagnosed (asymptomatic), using the classification developed by Achenbach in 1991 [31]. We understand internalizing symptoms as those manifested at a cognitive/internal level, such as anxiety, somatization, insecurity, fears, phobias, sadness, worry, obsessions, and mood instability. Diagnoses included in this category were adjustment disorders, anxiety disorders, depressive episodes, separation anxiety disorder, and obsessive-compulsive disorder (OCD). On the other hand, externalizing symptoms are those manifested behaviorally (externally) and characterized by poor emotional control; in this case, included diagnoses were attention-deficit hyperactivity disorder (ADHD), conduct disorders, and tics. Finally, mixed presentations were considered for those sharing both manifestations, such as autism spectrum disorders (ASD), intellectual disability, and psychosis. 

Information on psychological, socio-relational, and school functioning was collected through the GAF scale. The GAF scale is a tool used by mental health professionals to assess the progress of treatment and determine the severity of symptoms. This scale provides an objective measure of the severity of the patient’s mental condition on a continuum of health–illness [32]. The scoring is from 1 to 100 and can identify various ranges, indicating different levels of severity (Table 1). The GAF scale has an acceptable internal consistency, with Cronbach’s alpha values ranging from 0.70 to 0.90. Although it is no longer included in the DSM-5, it is highly relevant in clinical practice. It is validated and translated for the Spanish population [33,34].

### 2.4. Statistical Analysis

Descriptive analyses of the sample characteristics were conducted, presenting categorical variables in absolute frequencies and percentages and continuous variables in means and standard deviations (SD). The normality of the dataset was assessed using the Kolmogorov–Smirnov test.

To analyze the difference in values of the continuous variable GAF before the lockdown, three months after its end, and after three years, the Wilcoxon signed-rank test for related samples was applied, as the assumptions of normality were not met in some sample groups. Additionally, an analysis of covariance (ANCOVA) was performed to compare the means of the dependent continuous variable (GAF) between groups. Comparisons were made with the total sample and four population subgroups based on the diagnosis issued.

Statistical analysis was conducted using IBM SPSS Statistics version 28 (IBM Inc., Chicago, IL, USA). A significance level of *p* < 0.05 was considered indicative of statistical significance.

## 3. Results

The participants in the sample ranged in age from 3 to 18 years old and were predominantly males. Table 2 shows the number of participants in each of the groups according to their diagnostic category.

In Table 3, the impact of the pandemic has been analyzed by comparing the global functioning of children and adolescents at three different time points. Statistically significant differences are shown in the GAF scale scores between the scores obtained during the lockdown, three months after its end, and in the long term (three years after the end of the lockdown). It can be observed that the average symptom severity during the lockdown was 74.00, which increased to 80.52 three months later (transient symptoms and expected reactions to a psychosocial stressor). The difference reflects a significant improvement between both periods (Z = 12.050, *p* < 0.001), suggesting an overall improvement in the situation of patients treated in our unit. Similarly, long-term results also suggest statistically significant differences (Z = 16.237, *p* < 0.001), with the score rising to 88.76 points on the GAF scale (no or minimal symptoms). This once again indicates that patients treated in the unit experienced significant improvement following the intervention carried out in our unit after the COVID-19 pandemic. No significant differences were found between the scores of males and females at any of the evaluations, as detailed in Table 4.

When analyzing the scores obtained in each sample subgroup (depending on the diagnosed symptom category) during these periods (Table 5 and Table 6), it was observed that the average functioning of all patients significantly improved three months after the lockdown (*p* = 0.020) and in the long term, three years later (*p* < 0.001). Patients with internalizing, externalizing, and asymptomatic disorders showed a similar evolution of symptomatology severity throughout these three years: during lockdown, they presented transient symptoms reactive to psychosocial stress and finally remitted completely, indicating an overall satisfactory activity. Additionally, statistically significant differences were observed in all evaluation periods between the mixed pathology and the rest of the categories. The mixed pathology showed the least improvement compared to the other categories and also obtained the lowest scores in all periods: at the beginning of the lockdown, moderate dysfunction was shown, which evolved into milder symptomatology without reaching complete remission. Internalizing pathology showed the greatest improvement, both in the short and long term, followed by asymptomatic and externalizing pathology. The group of asymptomatic patients obtained the highest scores in all evaluations.

## 4. Discussion

The objective of this longitudinal study was to analyze the short-, medium-, and long-term evolution of the clinical status of children and adolescents treated in a CAMHS in the context of the COVID-19 pandemic.

In our sample, it is observed that the majority of evaluated patients met the criteria for externalizing symptoms such as ADHD or conduct disorders. This can be explained by the visible and disruptive nature of behavioral problems in childhood. Challenging behaviors, aggression, disobedience, tantrums, and poor academic performance are more evident and concerning for parents, motivating them to seek professional help to address these issues that affect family dynamics and the child’s development. [35]. Additionally, behavioral problems in children often generate more immediate concern due to their impact on the family and school environments, leading to more frequent consultations compared to other symptoms that may be less evident initially, such as anxiety or depression. [20,36,37].

The results of this study reflect a significant improvement in the clinical status of children and adolescents treated in a CAMHS during the COVID-19 lockdown period. This improvement was observed in all studied diagnostic groups and remained both in the short, medium, and long terms, with no significant differences observed between males and females.

Statistically significant differences were obtained in the average clinical status of all patients in the medium term, or three months after the lockdown (*p* = 0.020), and in the long term, or three years after (*p* < 0.001), showing improvement in all of them. However, it should be noted that, of all the analyzed patients, the diagnostic group that improved to a lesser extent in their functionality was the mixed presentations, where severe mental disorders are included (such as autism spectrum disorder, intellectual disability, and psychosis). This may be related to the fact that such conditions require specialized and continuous treatment, not improving solely with family support due to their complexity and chronic nature [38]. Similarly, it was the asymptomatic category that showed the highest scores in all evaluations.

Multiple studies have analyzed the immediate effects of the pandemic on the mental health of this population group. In most of them, an increase in levels of anxiety and depression in the child and adolescent population during the pandemic compared to previous periods was observed [20,21,22,23,24,25]. This impact was also evidenced in a meta-analysis that examined a total of 334 research studies addressing the subject; out of these, fourteen were included in the review, as they were the only ones that met the established criteria: original research studies, with a prospective methodological design, published from the year 2020 onwards, and whose results evaluated levels of depression, stress, anxiety, and/or behavioral problems using scales in the child and adolescent population during the SARS-CoV-2 pandemic [26]. The authors posited in their conclusions that internalizing symptoms (anxiety, depression, and emotional problems) significantly increased following the pandemic, in contrast to externalizing symptoms.

However, another study found contrary results, which found significant psychopathological effects of COVID-19 confinement [39]. This may be due to the previous treatment that this population was receiving in their inpatient specialized care unit. In other words, the study carried out a telephone follow-up of 422 children aged between 3 and 18 years during the months of March to May 2020 in the ESMI-J, recording their symptoms, diagnosis, and level of functioning using the GAF scale at each check-up visit. 

Such unexpected improvement could be related to a variety of factors, which can be grouped into three main categories: resilience, positive effects of lockdown, and therapeutic intervention received by patients. Regarding the first of these factors, the child and adolescent population has demonstrated a greater capacity to adapt to the lockdown situation than expected. This fact could be related to their cognitive flexibility, ability to cope with adversity, and the presence of social support networks [40]. 

Additionally, it is possible that in some cases, the presence of protective factors such as a positive family environment, high self-esteem, and the development of social skills may have contributed to such resilience. Regarding the positive effects of lockdown, it could be conjectured that the increased time spent with family during lockdown may have had a positive impact on the mental health of children and adolescents, as family interactions can provide emotional support, security, and a sense of belonging. Additionally, in some cases, the presence of protective factors such as a positive family environment, high self-esteem, and the development of social skills may have contributed to such resilience. Regarding the positive effects of lockdown, it could be conjectured that the increased time spent with family during lockdown may have had a positive impact on the mental health of children and adolescents, as family interactions can provide emotional support, security, and a sense of belonging [41]. 

On the other hand, a decrease in the pace of life and social obligations during lockdown may have allowed for a reduction in stress and an improvement in well-being, providing opportunities for personal development, such as exploring new hobbies, learning new skills, or strengthening creativity [42]. Additionally, regarding the third factor, the therapeutic intervention received by patients may have contributed to an improvement in their mental health. Psychological support can help children and adolescents manage their emotions, develop coping strategies, and improve self-esteem. The therapeutic strategies implemented during lockdown may have proven to be effective in improving the mental health of the pediatric population. Similarly, according to the consulted literature [20,43,44,45], one would expect that three months after lockdown, the mental health status of our patients would worsen reactively to the occurrence of a stressful life event. However, the results obtained in the present investigation are in opposition to this assertion.

These findings may contribute to understanding the resilience of pediatric mental health, as well as informing future interventions and policies. A high prevalence of mental disorders in children and adolescents can be observed, with long-term consequences impacting the quality of life, making early intervention essential. Early diagnosis and effective treatment are crucial. Child mental health should also be considered from a developmental perspective, understanding developmental milestones and their relation to observable behaviors. Additionally, mental health is not solely determined by biological factors; family, social, and educational environments significantly influence their psychological well-being; thus, interventions and policies should address these aspects comprehensively. Although not explicitly mentioned in the results, relevant theories such as attachment theory, cognitive development theory, and ecological theory are fundamental for understanding mental health in this population. This is an evolving field, and the current findings urge us to consider resilience from a holistic perspective, encompassing both individual and contextual factors [46,47,48].

It is essential to point out some limitations of the present study. Firstly, the sample consisted of children who attended a mental health service with mild symptomatologic involvement, so the results cannot be generalized to the entire pediatric population. Secondly, the degree of family exposure to the disease was low, with no deaths among the members of the families attended. This factor should be taken into account since it limits one of the main stressors of the situation. Thirdly, for ethical reasons, it was not possible to create a control group that would have allowed a comparison of the evolution. Additionally, the interaction between the described factors is complex, making it impossible to establish causal relationships between the variables. Therefore, further studies are necessary to determine which specific factors had a greater impact on improving mental health during lockdown. However, the results of this research are promising and suggest that the pediatric population may be more resilient to crisis situations than previously thought.

## 5. Conclusions

As conclusions of this study, it is emphasized that there was a significant improvement in the clinical status of children and adolescents treated at a CAMHS in the short, medium, and long term in the context of the COVID-19 pandemic. All analyzed groups obtained better scores in each of the evaluations conducted over time. However, no differences were observed according to the participants’ gender.

Furthermore, statistically significant differences were obtained between periods, depending on the diagnosis issued. Differences were observed between the mixed pathology and the rest of the categories, with the former showing the least improvement and also displaying the lowest scores. Internalizing pathology showed the greatest improvement, followed by the asymptomatic category, and finally, the externalizing pathology. Asymptomatic individuals had the highest scores in all evaluations.

The study’s conclusions suggest individualized and more intensive treatments for mixed pathologies, effective resource allocation, and detailed follow-ups. They also highlight the importance of developing specific protocols and training professionals. A continuous evaluation system can improve long-term clinical outcomes.

## Figures and Tables

**Table 1:** GAF scale.

100 91	Satisfactory activity in a wide range of activities, the evaluated child never seems overcome by the problems of his life and is valued by others because of his abundant positive qualities. No symptoms.
90 81	No or minimal symptoms (e.g., mild anxiety about an activity or situation), good activity in all areas, the child is interested and involved in a wide range of activities, with good relationships, cheerful and happy, with no worries or problems other than everyday ones.
80 71	If symptoms exist, they are transient and are expected reactions to psychosocial stressors; there is only a slight alteration in social or school activity.
70 61	Some mild symptoms (e.g., depressed mood and mild insomnia) or some difficulty in social, work, or school activity (e.g., missing a day’s homework or hiding an obligation).
60 51	Moderate symptoms (e.g., flattened affect and situational language, occasional distress attacks) or moderate difficulties in personal relationships or school activity (e.g., few friends, family conflicts, repeated problems with homework).
50 41	Severe symptoms (e.g., suicidal ideation, severe obsessive rituals) or any severe disturbance of personal relationships or school activity (e.g., isolated at home, unable to perform academic duties).
40 31	A disturbance in reality check or communication or significant disturbance in several areas such as schoolwork, family relationships, judgment, thinking, or mood (e.g., a child frequently hits younger brother or sister, is defiant, is totally disengaged from schoolwork).
30 21	Behavior is significantly influenced by delusions or hallucinations, or there is severe impairment of communication or judgment. Inability to function in almost all areas (e.g., stays in bed all day; no organized activity or relationships).
20 11	Some danger of causing injury to others or self (e.g., suicide attempts without overt expectation of death; frequently violent; manic excitement) or occasionally fails to maintain minimal personal hygiene (e.g., with fecal stains) or significant impairment of communication (e.g., very incoherent or mute)
10 1	Persistent danger of serious injury to others or self (e.g., recurrent violence) or persistent inability to maintain minimal personal hygiene or severe suicidal act with manifest expectation of death.
0	Inadequate information.

**Table 2:** Sample data.

Variables		*n* (420)	%
Age		11.83 ± 3.321	
Sex	Male	282	67.1
	Female	138	32.9
Diagnostic category	T. Internalizing	85	20.3
	T. Externalizing	176	41.9
	Mixed pathologies	109	25.9
	Asymptomatic	50	11.9

**Table 3:** GAF comparison during lockdown and three months after completion.

	Mean (*n* = 420)	SD	*p*-Value
GAF lockdown	74.00	11.95	<0.001
GAF three months later	80.52	13.15	
GAF three years later	88.76	12.86	

GAF: Global Assessment of Functioning; SD: Standard Deviation

**Table 4:** Differential scores of GAF values obtained during the lockdown and three months after its end based on gender.

	GAF Lockdown—GAF Three Months Later (*n* = 420)		GAF Lockdown—GAF Three Years Later (*n* = 420)	
	Mean differential score (SD)	Mean difference (SD)	Mean differential score (SD)	Mean difference (SD)
Male	6.790 (8.574)	−0.160 (0.933)	15.273 (10.030)	−0.372 (1.067)
Female	5.920 (10.194)	0.160 (0.933)	13.623 (12.453)	0.372 (1.067)
*p*-value	0.864		0.728	

GAF: Global Assessment of Functioning; SD: Standard Deviation.

**Table 5:** Mean GAF values obtained based on diagnostic category.

	GAF Lockdown (*n* = 420)	GAF Three Months Later (*n* = 420)	GAF Three Years Later (*n* = 420)	*p*-Value
T. Internalizing	75.47	82.92	92.07	<0.001 **
T. Externalizing	76.43	82.81	91.42	
Mixed pathologies	66.37	72.27	78.99	
Asymptomatic	79.58	86.28	95	

GAF: Global Assessment of Functioning ** *p* < 0.01.

**Table 6:** Differential scores of GAF values obtained during the lockdown and three months after its end based on diagnostic category.

Evaluation	Diagnostic Category	Mean Differential Score (SD)	Diagnostic Category	Means Difference (SD) (*n* = 420)	*p*	CI 95%		*p*-Value	Observed Power
						**LL**	**UL**		
GAF lockdown—GAF three months later	T. Internalizing	7.44 (10.348)	T. Externalizing	0.852 (1.162)	0.464	−1.433	3.137	0.020 **	0.757
			Mixed pathologies	3.658 (1.322)	0.006 **	1.060	6.256		
			Asymptomatic	−0.210 (1.576)	0.894	−3.307	2.888		
	T. Externalizing	6.36 (8.511)	T. Internalizing	−0.852 (1.162)	0.464	−3.137	1.433		
			Mixed pathologies	2.806 (1.142)	0.014 **	0.561	5.052		
			Asymptomatic	−1.061 (1.415)	0.454	−3.842	1.720		
	Mixed pathologies	5.90 (9.389)	T. Internalizing	−3.658 (1.322)	0.006 **	−6.256	−1.060		
			T. Externalizing	−2.806 (1.142)	0.014 **	−5.052	−0.561		
			Asymptomatic	−3.868 (1.589)	0.015 **	−6.990	−0.745		
	Asymptomatic	6.70 (8.636)	T. Internalizing	0.210 (1.576)	0.894	−2.888	3.307		
			T. Externalizing	1.061 (1.415)	0.454	−1.720	3.842		
			Mixed pathologies	3.868 (1.589)	0.015 **	0.745	6.990		
GAF lockdown—GAF three years later	T. Internalizing	16.60 (11.142)	T. Externalizing	1.222 (1.282	0.341	−1.298	3.742	<0.001 **	1.000
			Mixed pathologies	8.003 (1.457)	<0.001 **	5.138	10.868		
			Asymptomatic	−0.638 (1.738)	0.714	−4.054	2.778		
	T. Externalizing	14.93 (10.449)	T. Internalizing	−1.222 (1.282)	0.341	−3.742	1.298		
			Mixed pathologies	6.781 (1.260)	<0.001 **	4.305	9.257		
			Asymptomatic	−1.860 (1.560)	0.234	−4.927	1.207		
	Mixed pathologies	12.62 (12.578)	T. Internalizing	−8.003 (1.457)	<0.001 **	−10.868	−5.138		
			T. Externalizing	−6.781 (1.260)	<0.001 **	−9.258	−4.305		
			Asymptomatic	−8.641 (1.752)	<0.001 **	−12.085	−5.197		
	Asymptomatic	15.42 (6.895)	T. Internalizing	0.638 (1.560)	0.714	−2.778	4.054		
			T. Externalizing	1.860 (1.560)	0.234	−1.207	4.927		
			Mixed pathologies	8.641 (1.752)	<0.001 **	5.197	12.085		

GAF: Global Assessment of Functioning; SD: Standard Deviation; LL: lower limit; UL: upper limit; ** *p* < 0.01.

## Data Availability

Data supporting the conclusions of this study are available from the authors upon reasonable request.
